# Cumulative incidence and risk factors of myocardial infarction during 20 years of follow-up: comparing two cohorts of middle-aged men born 30 years apart

**DOI:** 10.1007/s00392-023-02308-y

**Published:** 2023-09-27

**Authors:** Maria Sakalaki, Aldina Pivodic, Kurt Svärdsudd, Per-Olof Hansson, Michael Fu

**Affiliations:** 1 Department of Molecular and Clinical Medicine, Institute of Medicine, Sahlgrenska Academy, University of Gothenburg, Sahlgrenska University Hospital/Östra Hospital, Gothenburg, Sweden; 2 Department of Medicine, Geriatrics and Emergency Medicine, Sahlgrenska University Hospital/Östra, Region Västra Götaland, Gothenburg, Sweden; 3 Department of Clinical Neuroscience, Institute of Neuroscience and Physiology, Sahlgrenska Academy, University of Gothenburg, Gothenburg, Sweden; 4 APNC Sweden, Gothenburg, Sweden; 5 Department of Public Health and Caring Sciences, Family Medicine and Preventive Medicine Section, Uppsala University, Uppsala, Sweden

**Keywords:** Prevention, Ischemic heart disease, Myocardial infarction

## Abstract

**Objective:**

To study cumulative incidence and predictors of myocardial infarction (MI) in two random general population samples consisting of middle-aged Swedish men born 30 years apart.

**Method:**

Results from the “*Study of Men Born In 1913”* and the *“Study of Men Born In 1943”*, two longitudinal cohort studies performed in the same geographic area and using the same methodology were compared. Both cohorts were followed prospectively from 50 to 70 years of age. MI was defined as first myocardial infarction, fatal or non-fatal.

**Results:**

Men born in 1943 had a 34% lower cumulative risk of first MI [HR 0.66 (0.50–0.88), *p* = 0.0051] during follow-up as compared to men born in 1913. Interaction analysis showed that hypertension had a significantly higher impact on risk of MI in cohort 1943 than in cohort 1913 [HR 2.33 (95% CI 1.41–3.83)] and [HR 1.10 (0.74–1.62)], *p* = 0.0009 respectively. The population attributable risk for hypertension was 2.5-fold higher in the cohort of men born in 1943 as compared to men born in 1913, and diabetes mellitus and sedentary lifestyle attributed more to MI risk in cohort 1943 than in cohort 1913. On the contrary, smoking and total cholesterol have less attributable risk to MI in cohort 1943 than in cohort 1913.

**Conclusion:**

Despite declining incident MI and improved cardiovascular prevention in general, hypertension remains an increasingly important attributable risk factor to MI together with diabetes mellitus and sedentary lifestyle over time.

**Supplementary Information:**

The online version contains supplementary material available at 10.1007/s00392-023-02308-y.

## Summary Points

**Table:** 

***What is already known on this topic***
Ischemic heart disease remains the leading cause of death globally, although decreasing levels of major cardiovascular risk factors.
Current smoking and cholesterol have declined, while obesity has increased.
***What this study adds?***
How different is the risk profile for incident myocardial infarction over time.
How much has the impact of different risk factors on MI changed over 30 years.
***How this study might affect research, practice, or policy?***
Influence how efforts should be dedicated in primary prevention to combat residual cardiovascular risk factors to further decrease incidence of MI.

## Introduction

Changes in cardiovascular risk factors through the past decades have previously been studied, showing generally lower levels of major risk factors, such as smoking and hyperlipidemia, but increasing rates of obesity [1–4]. In a Swedish male population, obesity at 50 years of age increased from 6.0% to 19.2% over a period of 50 years [1]. Simultaneously, mortality from cardiovascular disease (CVD) and incidence of myocardial infarction (MI) showed a decreasing trend in Sweden and other western countries [5–8]. We have also previously shown decline in all-cause mortality in the current study sample [9]. However, ischemic heart disease (IHD) remains the leading cause of death globally [10].

The decline of death rates from coronary heart disease most likely depends on a reduction of major risk factor levels, where lowering of total cholesterol has a considerable impact. Other contributing risk factors are decrease of smoking rates and blood pressure levels [11, 12]. Overweight and diabetes are established CHD risk factor, of which the increasing prevalence is alarming. Consequently, this circumstance has a diverse effect on CHD mortality as shown in the previous studies [11–13]. Furthermore, the PURE study investigated the effect of physical activity on CVD in 17 countries, showing that higher physical activity was associated with lower CVD events [14]. Simultaneously, low physical activity in high-income countries tends to increase [15].

This study aimed to examine changes in incidence rates and impact of various risk factors for incident MI during a 20-year follow-up period by comparing two randomly sampled cohorts of middle-aged men from the general population, born 30 years apart.

## Methods

### Study population

The “*Study of Men Born In 1913”* and the *“Study of Men Born In 1943”* are longitudinal cohort studies investigating cardiovascular risk factors and diseases. The cohort of men born in 1913 was a systematic sample drawn from the Swedish national population register, consisting of all men born in 1913 on a day divisible by 3 (the third, the sixth, and so on, of each month) and living in the city of Gothenburg. The criteria were fulfilled by 973 men, of whom 855 (87.9%) agreed to participate in the first health examination in 1963. All men were invited to re-examinations at ages of 54, 60, 67, 75, 80, and 100 [16].

The cohort of men born in 1943 was a random sample drawn from the Swedish national population register of 50% of all men who were born in 1943 and living in the city of Gothenburg. The criteria were fulfilled by 1463 men, of whom 798 (54.5%) agreed to participate in the examination in 1993. Follow-up examinations were performed at age 60 and 71 [17].

In the current study, only data from 20-year follow-up were included to enable comparison of both two cohorts, the “*Study of Men Born In 1913”* and the *“Study of Men Born In 1943”.*

### Examinations

Baseline and follow-up examinations followed the same study protocol for both cohorts. Data on smoking habits, alcohol consumption, leisure time physical activity, previous diseases, and pharmacological treatment were collected by questionnaires. Leisure time physical activity was assessed by the Saltin–Grimby Questionnaire [18] and coded as: 1 = sedentary (physically inactive); 2 = some light physical activity such as walking, riding a bicycle, or light gardening for at least 4 h per week; 3 = regular, moderate physical activity for a minimum of 3 h per week; and 4 = regular, vigorous physical training.

Smoking habits were measured by questionnaire and classified as 1 = never smoked, 2 = ex-smoker since 1 month or more, 3 = currently smoking 1–14 g/day, 4 = currently smoking 15–24 g/day and 5 = currently smoking 25 g/day or more. A cigarette was assumed to contain 1 g of tobacco, a cheroot 2 g, and a cigar 5 g.

Height and weight (in light indoor clothing) were measured and body mass index (BMI) ([weight in kg]/[height in m]^2^) was calculated. A standard cuff and a mercury manometer were used to measure blood pressure. Hypertension was defined as either current antihypertensive therapy or systolic blood pressure ≥ 140 mmHg, or diastolic blood pressure ≥ 90 mmHg. Fasting serum cholesterol levels were analyzed at the local accredited laboratory. Diabetes mellitus was diagnosed as elevated fasting plasma glucose or previously diagnosed and treated diabetes mellitus (pharmacological and/or non-pharmacological).

### Endpoint definition

MI was defined as first MI admitted to hospital, fatal or non-fatal. Information on MI was collected at each re-examination. Moreover, medical records were scrutinized to find and verify MI diagnoses for both cohorts. Data from The Swedish Hospital Discharge Register (since 1987) covering all hospital discharge diagnoses in Swedish residents, whether Swedish citizens or not, complemented the MI diagnosis for cohort 1943 and data from the Swedish Cause of Death Register, including all deaths in Sweden and abroad, for both cohorts. Diagnoses were identified by the International Classification of Diseases codes I21 (ICD 10) and 410 (ICD 8 and 9).

For 1913 cohort, only year of the incident non-fatal MI was available. July 1st was imputed for those cases. The exact dates of fatal MI for the 1913 cohort, as well as for fatal and non-fatal MI for the 1943 cohort were known and applied in the analyses. The 1913 cohort was followed from January 1st 1964 until the studied endpoint, death date or censoring at January 1^st^ 1984, whichever occurred first. The 1943 cohort was followed from January 1st 1994 until the studied endpoint, death date, date of visit in 2014 or January 1st 2014, whichever occurred first.

### Statistical analysis

Baseline characteristics were presented as number and percentage and mean ± standard deviation (± SD) as appropriate. For comparisons between the two cohorts, Fisher’s exact test was used for dichotomous variables, the Mantel–Haenszel chi-square trend test for ordered categorical variables and the Mann–Whitney *U* test for continuous variables.

Sedentary lifestyle, and never-smokers or ex-smoker were used as the reference in the comparisons for the respective variable. The risk factors were studied at study baseline and time-updated at 10-year scheduled visits, 1973 visit for the 1913 cohort and 2003 visit for the 1943 cohort. Presence of diabetes mellitus was studied time-updated at the date of incidence for 1943 cohort, and on July 1st for the year of onset for the 1913 cohort, since only to the year of onset was available in this data set.

Crude event rates were calculated as number of events divided by follow-up time and expressed as per 1000 person-years. The 95% confidence intervals (CI) were estimated using exact Poisson limits. The importance of risk factors on MI incidence was studied applying univariable time-updated Cox´s proportional hazards models and population attributable risk.

Hazard ratios (HR) in Cox´s proportional hazards models were presented as effect measurement together with their 95% CI. The proportional hazard was satisfied for all variables except for smoking that was additionally analyzed for the periods 0–10 years and 10–20 years of follow-up to describe the changes in HRs as a result of significant interaction with time. The importance of various exposure variables on MI between the two cohorts was studied by including an interaction term between the variable of interest and the cohort in the Cox´s regression.

Beside unadjusted analyses, the Cox hazard models for prediction of myocardial infarction were additionally adjusted for risk factors hypertension, diabetes mellitus, BMI, sedentary lifestyle, smoking, and cholesterol.

Population attributable risk was assessed by calculating the difference of the incidence of MI in the exposed versus non-exposed group for a specific risk factor. The following binary exposure variables were studied: SBP ≥ 140 mmHg, DBP ≥ 90 mmHg, hypertension, BP medication, diabetes, smoking, weight ≥ 80 kg, BMI ≥ 25 kg/m^2^, sedentary lifestyle, cholesterol ≥ 6.2 mmol/l, and cholesterol ≥ 5.0 mmol/l.

Graphical presentation of the cumulative incidence of myocardial infarction accounted death due to other causes than MI as competing risk. All tests were two-tailed and the level of significance was set at *p* < 0.05. Statistical analyses were performed with SAS statistical software V.9.4 (SAS Institute Inc., Cary NC, USA).

## Results

### Study cohorts

In the cohort of men born in 1913, ten (1.2%) participants were excluded because of MI before the baseline examinations and three (0.4%) participants due to death before the age of 51 years leaving a study population of 842 participants in this cohort. For men born in 1943, 15 (1.9%) participants were excluded due to MI before baseline, leaving 783 participants as study populations in this group for follow-up.

### Cumulative incidence of MI between the two cohorts

**Fig. 1:**
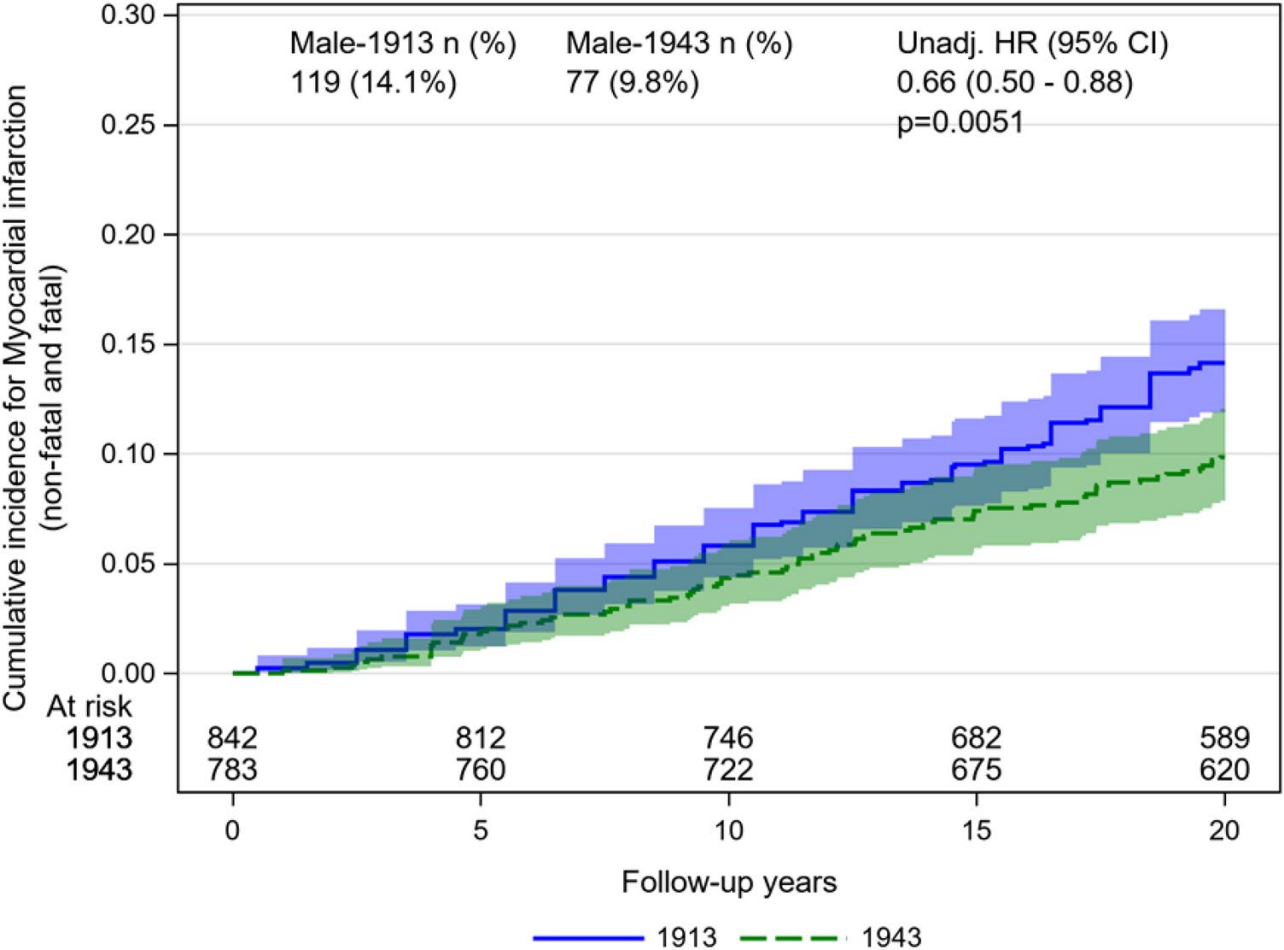
Cumulative incidence for myocardial infarction in men born 1913 and 1943. Other death than fatal MI accounted as competing risk. *HR*  hazard ratio

During follow-up 119 men born in 1913 developed MI (14.1%, event rate 8.04 per 1000 person-years [95% CI 6.72–9.62]) as compared to 77 participants among men born in 1943 (9.8%, event rate 5.4 per 1000 person-years [95% CI 4.30–6.72]). Unadjusted, men born in 1943 had a 34% lower risk of MI [HR 0.66, 95% CI (0.50–0.88), *p* = 0.0051] as compared to men born in 1913 during follow-up (Fig. 1).

### Distributions of risk factors comparing the two cohorts

Table 1 illustrates cardiovascular risk factors for men born in 1913 and 1943 at study baseline and follow-up presented for men with or with no MI within 20 years of follow-up. In general, a significant trend of improved risk modification was observed in the more recent cohort as decreased blood pressure, fewer incident hypertension, increased blood pressure-lowering medications, success in smoking cessation, declined lipid level, and increased physical activities. However, diabetes mellitus, body weight, and BMI kept increasing.

**Table 1:** Patient data of potential cardiovascular risk factors for men born in 1913 and 1943 at study start, and follow-up examinations, presented for men with or without MI within 20 years of follow-up

	Myocardial infarction					
	No myocardial infarction during 20-year follow-up			Myocardial infarction during 20-year follow-up		
	1913 born men *n* = 723	1943 born men *n* = 706	*p* value	1913 born men *n* = 119	1943 born men *n* = 77	*p* value
Systolic blood pressure (mmHg) at start	137.1 (19.7)	128.1 (17.0)	< .0001	144.7 (26.1)	133.4 (18.1)	0.0038
Systolic blood pressure (mmHg) at 10y follow-up	146.5 (23.4)	143.4 (19.3)	0.077	149.3 (26.9)	148.7 (22.5)	0.97
Diastolic blood pressure (mmHg) at start	90.9 (12.8)	84.1 (10.5)	< .0001	95.7 (14.4)	86.9 (11.6)	< .0001
Diastolic blood pressure (mmHg) at 10y follow-up	89.3 (13.0)	85.1 (10.3)	< .0001	90.9 (15.2)	87.1 (11.7)	0.14
Hypertension at start	486 (67.2%)	261 (37.0%)	< .0001	85 (71.4%)	45 (58.4%)	0.086
Hypertension at 10y follow-up	428 (69.6%)	376 (64.9%)	0.099	66 (68.8%)	51 (79.7%)	0.18
Blood pressure-lowering medication at start	9 (1.2%)	33 (4.7%)	0.0002	4 (3.4%)	14 (18.2%)	0.0012
Blood pressure-lowering medication at 10y follow-up	56 (9.5%)	109 (18.8%)	< .0001	23 (26.4%)	29 (46.0%)	0.021
Diabetes at start	10 (1.4%)	15 (2.1%)	0.39	3 (2.5%)	7 (9.1%)	0.091
Diabetes at 10y follow-up	37 (5.1%)	36 (5.1%)	1.00	11 (9.2%)	8 (10.4%)	0.97
Diabetes during 20y follow-up	64 (8.9%)	88 (12.5%)	0.033	17 (14.3%)	20 (26.0%)	0.065
Smoker at start	386 (53.4%)	210 (29.7%)	< .0001	84 (70.6%)	33 (42.9%)	0.0002
Smoker at 10y follow-up	248 (41.3%)	84 (14.5%)	< .0001	43 (46.7%)	14 (21.9%)	0.0023
Weight (kg) at start	75.8 (10.9)	83.3 (12.0)	< .0001	77.0 (11.4)	84.5 (12.9)	< .0001
Weight (kg) at 10y follow-up	78.4 (11.1)	85.9 (12.8)	< .0001	79.8 (11.9)	89.0 (13.1)	< .0001
Body mass index (kg/m2) at start	24.7 (3.2)	26.1 (3.4)	< .0001	25.0 (3.2)	26.7 (3.1)	0.0002
Body mass index (kg/m2) at 10y follow-up	25.5 (3.3)	26.9 (3.7)	< .0001	25.7 (3.5)	28.2 (3.6)	< .0001
Sedentary lifestyle at start	254 (35.8%)	105 (14.9%)	< .0001	37 (32.2%)	17 (22.1%)	0.17
Sedentary lifestyle at 10y follow-up	135 (22.8%)	63 (10.9%)	< .0001	21 (22.8%)	10 (15.6%)	0.37
Cholesterol (mmol/l) at start	6.33 (1.11)	5.84 (1.03)	< .0001	6.66 (1.05)	6.19 (1.05)	0.0005
Cholesterol (mmol/l) at 10y follow-up	6.50 (1.20)	5.44 (0.90)	< .0001	6.71 (1.09)	5.05 (1.02)	< .0001

For categorical variables *n* (%) is presented

For continuous variables Mean (SD) is presented

Treatment of hypertension had become better in the cohort of men born in 1943 as compared to the 1913, but only a small proportion of patients have received blood pressure-lowering modifications. Moreover, hypertension was rapidly increasing from 37.0% to 64.9% in those with no MI, and from 58,4% to 79,7% in those with MI in cohort 1943 from baseline to 10 year follow-up, while in cohort 1913, the percentage of individuals with hypertension was about 70% for both groups at baseline and at 10 years.

### Impact of risk factors on myocardial infarction in the two cohorts

**Fig. 2:**
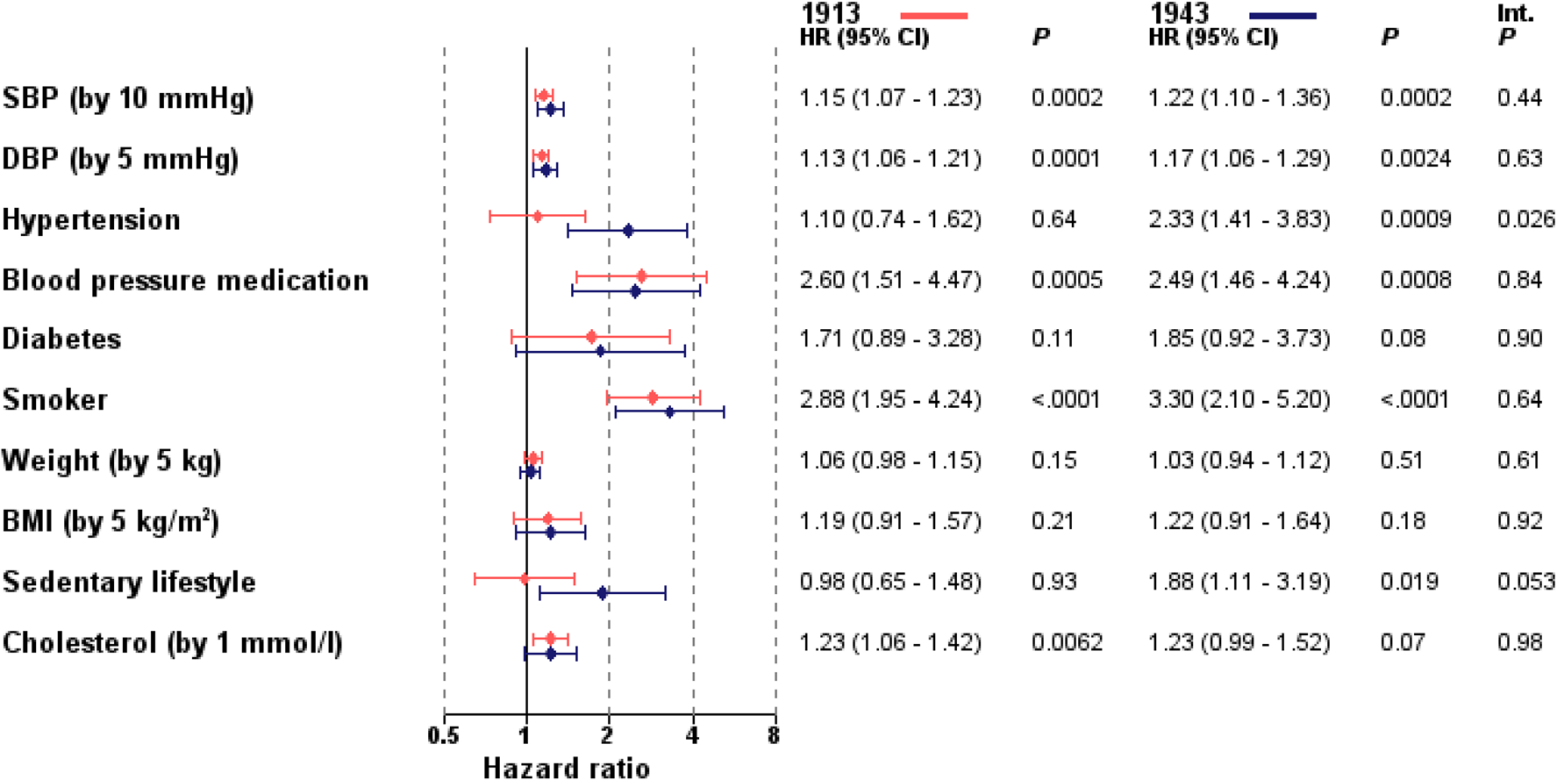
Univariable Cox proportional hazards models for prediction of myocardial infarction during 20 year follow-up and the interaction effect of risk factors among two cohorts of men born in 1913 and 1943

Figure 2 demonstrates the Cox´s hazard models for prediction of incident MI during 20 year follow-up and the association with different risk factors, showing differences between the two cohorts. Elevated systolic blood pressure, elevated diastolic blood pressure, and treatment with blood pressure-lowering medications were all associated with increased risk for developing MI in both cohorts. Hypertension was significantly associated with the risk of myocardial infarction in the 1943 cohort, but not in the 1913 cohort. Smoking was almost equally associated with risk for MI in both cohorts (HR 2.88 (95% CI 1.95–4.24), *p* < 0.0001 for cohort 1913, vs HR 3.30 (95% CI 2.10–5.20), *p* < 0.0001 for cohort 1943). However, in the 1913 cohort, the proportional hazard assumption was not fulfilled, shown by the significant interaction for smoking*log(time), with a negative parameter estimate. For the time period 0–10 years, the HR for smokers vs non-smokers was 7.51 (95% CI 3.38–16.73), and for 10–20 years, it was 1.77 (95% CI 1.10–2.85).

Despite numerically important differences, no significant impact on developing MI was observed for either of the two cohorts regarding diabetes mellitus or BMI. Sedentary lifestyle was associated with an increased MI risk in the cohort of men born in 1943 (HR 1.88, 95% CI 1.11–3.19, *p* = 0.019) but not in the 1913 cohort (HR 0.98, 0.65–1.48, *p* = 0.93). Cholesterol had similar association to MI in the two cohorts, but statistically non-significant in men born 1943 (HR 1.23 per 1 mmol/l increase, 95% CI 0.99–1.52, *p* = 0.067) and significant for the 1913 cohort (HR 1.23 per 1 mmol/l increase, 95% CI 1.06–1.42, *p* = 0.0062).

Similar results were found on the impact of developing MI for each risk factor after adjusting for hypertension, diabetes mellitus, BMI, sedentary lifestyle, smoking, and cholesterol (Supplementary material).

Interaction analysis showed that hypertension has significantly higher impact as risk factor to MI in the cohort 1943 than in the cohort 1913. A similar trend was found for sedentary lifestyle but without statistical significance (*p* = 0.053).

### Population attributable risk of MI, in the two cohorts

Attributable risk analysis showed that hypertension including those treated with blood pressure-lowering medications has significantly higher attributable risk to MI, 2.5-fold higher in cohort 1943 than cohort 1913, Fig. 3. Among those treated with blood pressure-lowering medications, the proportion of attributable risk in both cohorts was greater (21.2% in cohort 1943 and 16,9% in cohort 1913); however, the magnitude of augmentation of attributable risk is relatively smaller between the two cohorts.

**Fig. 3:**
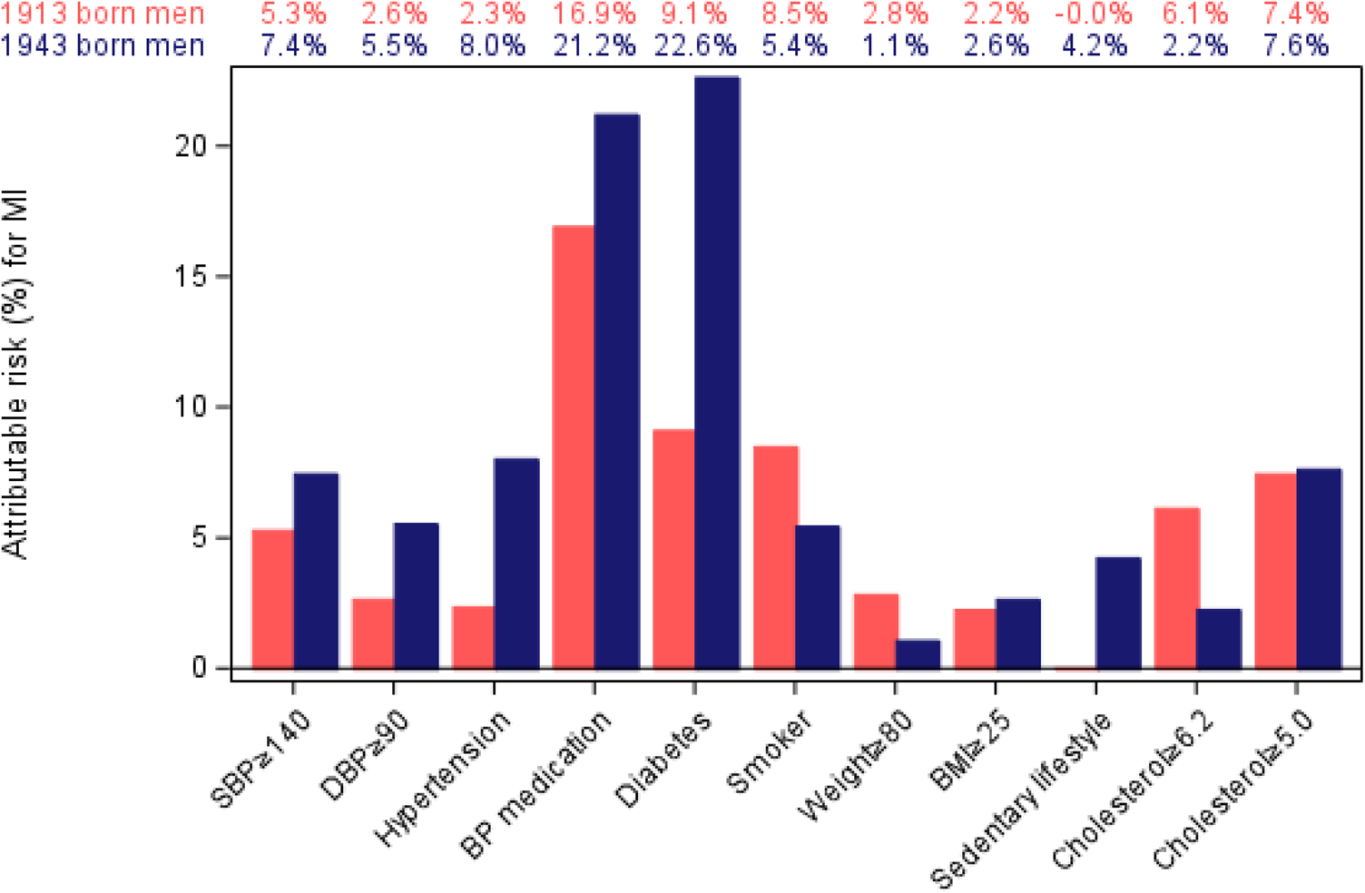
Population attributable risk of risk factors for myocardial infarction in two cohorts of men born in 1913 and 1943

Diabetes was the risk factor contributing most to MI incidence in the group of men born 1943 (22.6%) and second most in the cohort of men born 1913 (9.1%). Sedentary life did not contribute to MI incidence in men born 1913, while it contributed with 4.2% in men born 1943. Smoking and total cholesterol had less attributable risk to MI in cohort 1943 than cohort 1913.

## Discussion

This study showed a decreased incidence of MI in the groups compared. Furthermore, hypertension remains an increasing important attributable risk factor to MI together with diabetes mellitus and sedentary lifestyle over calendar time.

Despite a marked decrease in all-cause mortality compared with cohort 1913 [9], still around 10% of the men in the cohort 1943 developed MI between the ages of 51 and 71 years. We sought the reason of this by exploring underlying risk factors.

Hypertension was associated with more than a doubled risk of MI among men born in 1943 than those born in 1913, notwithstanding that treatment for hypertension has increased. However, treatment with blood pressure-lowering medication does not necessarily imply optimally achieved treatment goal. As a matter of fact, treatment goals were often not met, and therefore, risk reduction by treatment has not been fully achieved [19].

Likewise, among those with incident MI, hypertension occurred more frequently in cohort 1943 from 58% at baseline to 80% 10 years later, in contrast to the fact that hypertension was present almost to a similar extent in cohort 1913 between baseline and upon 10 years of follow-up. Moreover, no difference in blood pressure levels was found between the two cohorts at 10 years of follow-up, although blood pressure-lowering medication increased from 26 to 46% during the 30 years apart. Our data were in line with the previous studies in Europe and the US [20–24]. There might be several reasons for uncontrolled hypertension, as age, lack of knowledge of appropriate control of systolic blood pressure, multi-drug therapy and having side-effects of medication [25], and sedentary behavior might be another [22].

Finally, hypertension contributed to MI incidence to a greater extent in the 1943 cohort compared to men born in 1913. One of the explanations is more rapidly increased incident hypertension and an increased mean blood pressure in cohort 1943 compared to the 1913 cohort, despite increased blood pressure-lowering medications.

Diabetes mellitus contributed to MI incidence in both cohorts, but the presence of diabetes mellitus was not a significant risk factor in this report, possibly due to the fact that the two cohorts were still rather young, and few had developed diabetes. This was supported by earlier publications based on a time-dependent updated analysis of the cohort of men born in 1913 followed until 80 years of age, in which diabetes mellitus has proved to be a strong risk factor for MI.

It is known that many risk factors for MI interact with each other, for example obesity and sedentary lifestyle, can affect the development of type-II diabetes mellitus. In our study, the adjusted impact of risk factors on developing MI was similar to that in the unadjusted analyses. For instance, the impact of hypertension and diabetes on CVD outcomes are independent of BMI, sedentary lifestyle, and other risk factors. However, these risk factors may have additive impact on the progression of CVD outcome.

A sedentary lifestyle was reported less frequently, and regular or hard exercise increased during follow-up among men born in 1943. Similar findings were seen in other studies [4, 26]. Furthermore, in our analysis, a sedentary lifestyle was found to increase the risk of having an event of MI in the group of men born 1943 and the relevance of this risk factor seems to have increased as compared to the group of men born in 1913. The Swedish community has like many other countries undergone a computerization. One might speculate that even though stating no regular exercise as a participant born in 1913, daily life may have been different among these men, including more occupational activity. This might explain our findings among men born 1913, although reporting a sedentary lifestyle these men were still physically active. However, the association of occupational activity with MI and IHD has been inconsistent [27–29]. Held et al. showed in their study that light-to-moderate occupational physical activity had a lower risk of MI when compared to a sedentary lifestyle, while heavy work did not [29]. Nevertheless, the lack of physical activity in leisure time as a risk factor of MI is well known [30], and interventions for increasing physical activity in primary prevention are warranted.

Being a current smoker was associated with the highest risk of MI during 20 years of follow-up in both cohorts with similar increased risk, and hence, no difference was found between the two cohorts.

Finally, higher cholesterol was significantly associated with higher incidence of MI only in men born 1913. This might be attributable to lower variability in cholesterol levels in the 1943 cohort, due to more effective lipid lowering medication among men born in 1943.

Incident MI remained high in a contemporary era despite its incidence was significantly declining as compared with 30 years previously. Moreover, hypertension, diabetes mellitus, and a sedentary lifestyle were identified to become increasingly important contributing risk factors to MI. Therefore, our results highlight the questions of how to optimally allocate resources to further target these risk factors and further decrease incident MI in the future.

### Strengths and limitations

The fairly small sample size is one limitation and only men were included making this cohort less generally representative. Another limitation is that we cannot strictly compare the occurrence of MI events between the two cohorts, because they were studied 30 years apart and diagnostic practices including biochemical marker have been adjusted.

The strengths of the current study are that we analyzed systematic and random samples from the general population, all the same sex, age, and living in the same geographic area. The two cohorts were established using the same protocol and part of our research staff being the same. Finally, both cohorts were followed for 2 decades, which not only enable time-updated analysis but also provide a high number of person-years with more than 10,000 person-years of exposure.

## Conclusion

The cumulative incidence of MI has decreased, and despite improved cardiovascular prevention in general, hypertension remains an increasingly important attributable risk factor to MI together with diabetes mellitus and sedentary lifestyle over time. Impacts of cardiovascular risk factors for MI have changed and greater efforts should be dedicated in primary prevention to combat these residual risk factors.

## Supplementary Information

Below is the link to the electronic supplementary material.

Supplementary file1 (RTF 83 KB)

## Data Availability

The dataset supporting the conclusions of this article are included within the article. The datasets during and/or analyzed during the current study are available from the corresponding author on reasonable request.

## Declarations

### Patient and public involvement

Patient and/or public were not involved in the design, conduct, reporting, or dissemination plans of this research.

### Provenance and peer review

Not commissioned, externally peer reviewed.
