# Supplementary material for: Cumulative incidence and risk factors of myocardial infarction during 20 years of follow-up: comparing two cohorts of middle-aged men born 30 years apart
Source: Clin Res Cardiol. 2023 Sep 27;113(12):1661–9. doi: 10.1007/s00392-023-02308-y (PMC11579158; doi:10.1007/s00392-023-02308-y)
Supplement: Supplementary file 1 — Supplementary file1 (RTF 83 KB) [file 392_2023_2308_MOESM1_ESM.rtf]

Adjusted Cox proportional hazards models for prediction of myocardial infarction within 20 years of follow-up for 51 years old men and their risk factors at 50 years of age and during the follow-up among 1913 and 1943 cohorts.

	1913 cohort	1943 cohort	
Time-updated predictor (at start and 10 years data or event date for diabetes)	Value/Comparison	HR (95% CI)	p-value	HR (95% CI)	p-value	
Hypertension	Yes vs No	1.01 (0.66 - 1.53)	0.97	2.24 (1.34 - 3.74)	0.0022	
BMI (kg/m2)	by 5 kg/m2 increase	1.21 (0.90 - 1.62)	0.20	1.02 (0.75 - 1.39)	0.90	
Sedentary lifestyle	Yes vs No	0.98 (0.65 - 1.47)	0.91	1.75 (1.03 - 3.00)	0.040	
	
Diabetes	Yes vs No	1.61 (0.83 - 3.13)	0.16	1.66 (0.81 - 3.39)	0.16	
BMI (kg/m2)	by 5 kg/m2 increase	1.18 (0.89 - 1.55)	0.25	1.13 (0.84 - 1.52)	0.43	
Sedentary lifestyle	Yes vs No	0.96 (0.64 - 1.45)	0.85	1.77 (1.03 - 3.02)	0.038	
	
Hypertension	Yes vs No	1.02 (0.67 - 1.56)	0.92	2.40 (1.43 - 4.01)	0.0009	
Diabetes	Yes vs No	1.61 (0.83 - 3.11)	0.16	1.58 (0.77 - 3.22)	0.21	
BMI (kg/m2)	by 5 kg/m2 increase	1.27 (0.95 - 1.71)	0.11	1.01 (0.73 - 1.39)	0.96	
Sedentary lifestyle	Yes vs No	0.94 (0.62 - 1.42)	0.77	1.50 (0.87 - 2.59)	0.14	
Smoker	Yes vs No	3.03 (2.04 - 4.50)	<.0001	3.38 (2.14 - 5.35)	<.0001	
Cholesterol (mmol/l)	by 1 mmol/l increase	1.21 (1.03 - 1.42)	0.018	1.24 (1.00 - 1.54)	0.054	
	
